# Alterations in Cortical Activation among Soccer Athletes with Chronic Ankle Instability during Drop-Jump Landing: A Preliminary Study

**DOI:** 10.3390/brainsci12050664

**Published:** 2022-05-19

**Authors:** Xiaoya Zhang, Wanrongyu Su, Bin Ruan, Yu Zang

**Affiliations:** School of Sports Medicine and Rehabilitation, Beijing Sport University, Beijing 100084, China; hanbingxy@sina.com (X.Z.); 15517966968@163.com (W.S.); ruanbing@bsu.edu.cn (B.R.)

**Keywords:** chronic ankle instability, electroencephalography, cortical activation, power spectral density, soccer athletes

## Abstract

Background: Chronic ankle instability (CAI) is a common peripheral joint injury and there is still no consensus on the mechanisms. It is necessary to investigate electrocortical parameters to provide clinical insight into the functional alterations of brain activity after an ankle sprain, which would greatly affect the implementation of rehabilitation plans. The purpose of this study was to assess cortical activation characteristics during drop-jump landing among soccer athletes with CAI. Methods: A total of 24 participants performed the drop-jump landing task on a force platform while wearing a 64-channel EEG system. The differences of power spectral density (PSD) in theta and alpha (alpha-1 and alpha-2) bands were analyzed between two groups (CAI vs. CON) and between two limbs (injured vs. healthy). Results: CAI participants demonstrated significantly higher theta power at the frontal electrode than that in healthy control individuals (F_(1,22)_ = 7.726, *p* = 0.011, η^2^p = 0.260). No difference in parietal alpha-1 and alpha-2 power was found between groups (alpha-1: F_(1,22)_ = 0.297, *p* = 0.591, η^2^p = 0.013; alpha-2: F_(1,22)_ = 0.118, *p* = 0.734, η^2^p = 0.005). No limb differences were presented for any frequency band in selected cortical areas (alpha-1: F_(1,22)_ = 0.149, *p* = 0.703, η^2^p = 0.007; alpha-2: F_(1,22)_ = 0.166, *p* = 0.688, η^2^p = 0.007; theta: F_(1,22)_ = 2.256, *p* = 0.147, η^2^p = 0.093). Conclusions: Theta power at the frontal cortex was higher in soccer athletes with CAI during drop-jump landing. Differences in cortical activation provided evidence for an altered neural mechanism of postural control among soccer athletes with CAI.

## 1. Introduction

Ankle sprain, the most common lower extremity injury, accounting for 9.4% to 18% of all sports injuries [1,2,3], often occurs in walking, running, jumping, and cutting movements [4]. It has been reported that at least 73% of individuals with a history of ankle sprain had some type of residual symptoms [5], and among which chronic ankle instability (CAI) was the most frequent sequelae [6]. Researchers concluded that within the first year after an initial ankle sprain, 40% of patients developed into CAI [7], which is characterized by recurrent sprains, pain, perceived instability, and dysfunction [8,9], leading to 20–40% of individuals having to stop exercising [10]. Soccer, as the most popular sport in the world, is a complex contact sport that involves relatively high risks and rates of injury in both professional and amateur players [11,12]. In addition, the ankle joint is under the most stress due to sudden postural changes and stops, jumping, and tackling [13]. Techniques such as tackling can create a corresponding eversion or inversion rotation of the ankle, thus increasing the risk of ankle sprain [14], which not only reduces quality of life and impairs athletic performance, but also increases the risk of joint degeneration and early-onset osteoarthritis. According to statistics, the incidence of osteoarthritis in CAI people is 68–75% [15], significantly higher than that in general population [16], and this prevalence of osteoarthritis is higher in soccer players with CAI [17,18], which brings great pain to patients and also imposes a considerable burden on health and economic sectors. Therefore, exploring the mechanism of CAI in soccer players can provide a theoretical basis for prevention and rehabilitation, so as to reduce ankle sprains and shorten suspension time.

Despite the theories regarding the development of CAI evolving over decades, there is still no consensus on the mechanisms of CAI. Most traditional models believe that CAI is a musculoskeletal disorder with adaptations typically occurring in the periphery rather than in the brain [19]. However, recent studies [20,21,22,23,24] have continuously confirmed that changes in the central nervous system (CNS) may occur after acute and chronic ligament injury, and these changes involved multiple cortical regions in the brain, such as the somatosensory cortex and motor cortex, which may negatively affect the recovery process and be associated with self-reported function [19]. As a result, one widely held view is that impaired sensorimotor integration through complex neural networks contributes to the development of CAI [25]. That is, with deficient ligament afferent, proprioception, and other somatosensory input, the brain is less able to modulate movement and joint stability through descending neuromuscular pathways [26]. Reorganization of the CNS treated the ankle sprain injury as a neurophysiological dysfunction rather than as a simple peripheral musculoskeletal injury [27], and this evidence explained the severe and long-standing dysfunction associated with CAI [28]. 

Research at the reflexive and cortical levels of CNS adaptations in individuals with ligament injury has grown rapidly [29,30,31]. However, studies on the ankle are significantly less prevalent than those on the knee. The earliest study on cortical plasticity after joint injury in humans was using electroencephalography (EEG) to observe changes in the somatosensory cortex in an anterior cruciate ligament (ACL) injury population [32]. This neurophysiological technique uses surface electrodes to detect electrophysiological signals from excitatory and inhibitory action potential depolarization in various areas of the cerebral cortex. Therefore, using EEG to assess cortical activation can provide insight into CNS reorganization by highlighting areas of the brain that may be associated with people who have sports injuries. Unfortunately, there are only three EEG studies [33,34,35] on CAI or functional ankle instability (FAI) individuals, and only one of those studies focused on athletes. In the studies mentioned above, researchers aimed at investigating the effects of training program on cortical activities. In addition, the existing literature has not yet presented a clear understanding of cortical changes that exist among the CAI population. Thus, continued investigation of cortical activity may help to explain how neuromuscular impairments arise. Meanwhile, it is crucial to understand the neuromechanical alterations after an ankle sprain in order to aid the secondary prevention of their long-term sequelae and make advances in therapeutic treatments [19]. Therefore, the purpose of this study was to assess cortical activation characteristics in soccer athletes with CAI during drop-jump landing movement. Based on previous study results [29] and given that landing tasks are more challenging for people with CAI, which means that this group of individuals requires more attention and neural resources, we hypothesized that CAI participants would display greater cortical activation than heathy control participants (CON) while they were performing the drop-jump landing task. 

## 2. Methods

### 2.1. Study Design

This report is a part of larger explorative investigation of cortical activity among soccer athletes with CAI. In order to examine characteristics in central activation patterns in the CAI population, the current study carried out a groups (CAI vs. CON) × limbs (injured vs. healthy) design. All tests were performed on both injured (also known as involved) and healthy (also known as uninvolved) side and comparisons were made between groups and limbs. Sample size was calculated using G*Power software (v.3.1) with 80% power, 5% type I error, and 55% effect size, and a minimum of 10 participants in each group were recommended [36].

### 2.2. Participants

A total of 26 volunteers were enrolled in this study. Two potential subjects in the CON group were excluded, one due to failure EEG recording, the other due to COVID-19 prevention and control. As a result, 24 participants were included in data analysis, and there were no differences in demographics between groups except CAIT score (t = −18.534, *p* = 0.000) (Table 1). Inclusion criteria for CAI participants were as follows: (1) being a collegiate soccer athlete; (2) being between 18 and 24 years of age; (3) unilateral injured; (4) a history of at least one significant lateral ankle sprain, including inflammatory symptoms and disruption of sport or physical activity; (5) the initial sprain and the most recent injury must have occurred more than 12 and 3 months prior to the study enrollment, respectively; (6) a history of at least 2 episodes of ‘giving way’, and/or recurrent sprain and/or ‘feelings of instability’ in the 6 months prior to the study enrollment; and (7) a CAIT score ≤ 24. All but the first three meet the criteria as recommended by the International Ankle Consortium for CAI patients [9]. Inclusion criteria for healthy control participants were (1) being a collegiate soccer athlete; (2) being between 18 and 24 years of age; (3) without history of ankle sprain; and (4) meeting a CAIT score ≥ 28. The involved limb for CON subjects was matched by limb dominance to the CAI group. Volunteers were excluded if they (1) had a history of lower limb surgery or fracture; (2) had an acute injury to the musculoskeletal structures of other joints of the lower extremity in the previous 3 months, which impacted joint integrity and function; (3) had a history of a balance or vestibular disorder; (4) had any other health problem that may have affected their balance; or (5) had a positive result in the talus tilt test and/or anterior ankle drawer test.

A single investigator (Y.Z.) performed all the screening procedures for enrolling participants. All participants had normal vision at the time of the experiment and were instructed to refrain from consuming caffeine within 12 h before the EEG test started. Before experiment, the subjects were informed in detail about the procedure and provided written informed consent. This study was approved by the Sports Science Experiment Ethics Committee of Beijing Sport University (2021195H) and registered in Chinese Clinical Trial Registry (ChiCTR2200055738).

### 2.3. Electroencephalography Test Protocol

The test was conducted at the Sport Rehabilitation of Beijing Sport University. Given that this study was part of a larger investigation, the task was evaluated while the athletes were on the force platform (Kistler.9286A, Switzerland). EEG data were collected using 64 Ag/AgCl scalp electrodes (NeuSen.W64, Neuracle, Changzhou, China) placed in accordance with the international 10/20 system. Cortical activity was recorded continuously via 16 EEG channels (FP1, FP2, F3, Fz, F4, FC3, FCz, FC4, C3, Cz, C4, CP3, CP4, P3, Pz, P4), and each electrode impedance was kept lower than 10 KΩ [37,38] throughout the experiment to ensure a sufficient signal-to-noise ratio. The acquired EEG signals were sampled at 1000 Hz by the NeuroNexus on a dedicated computer and saved for offline analysis. 

Cortical activity was measured while subjects performed the forward drop-jump landing task on the force platform [39]. To reduce bias, all participants wore the same shoes and were tested with random legs order. On oral cue, the players took off with 2 feet and landed on the testing leg from an aerobic step of 20 cm height. They were asked to balance for 10 s with their hands on the hips, opposite hip flexed to 30°, and knee flexed to 45°, while keeping their eyes fixed on a visual target on the wall. Both legs were tested 3 successful times with trials separated by 30 s of rest [40]. One practice trial per leg was performed before actual testing commenced. No instructions were given for jump height. Participants were asked to keep their eyes open and blink comfortably throughout the testing. A trial in which the participant did not maintain balance for the full required time, moved hands off hips, hopped or shifted the involved foot on the force plate, touched down with the contralateral leg, or landed with the involved foot not completely on the force plate was considered a failure. The test was stopped if participants failed, and the trial was reattempted until 3 successful trials were completed. The entire test was supervised by two investigators (X.Z. and W.S.)

### 2.4. Data Reduction and Analysis

**Figure 1:**
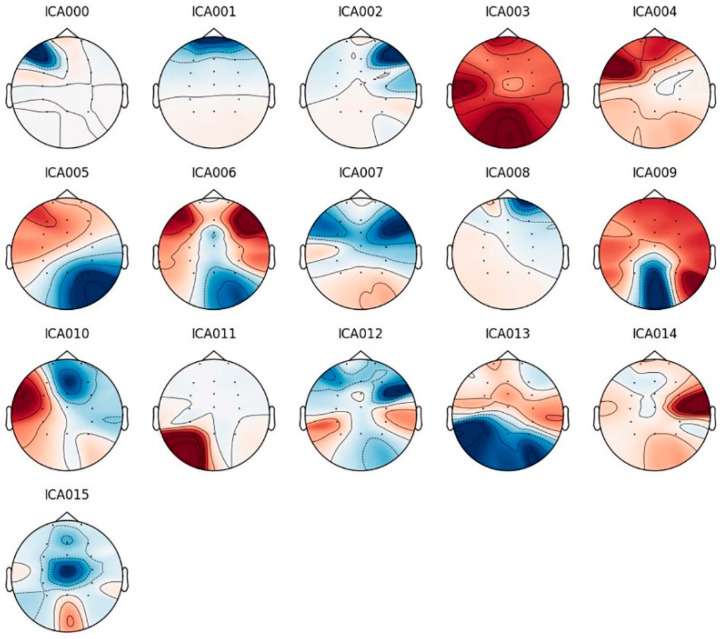
Topographic map of ICA components.

**Figure 2:**
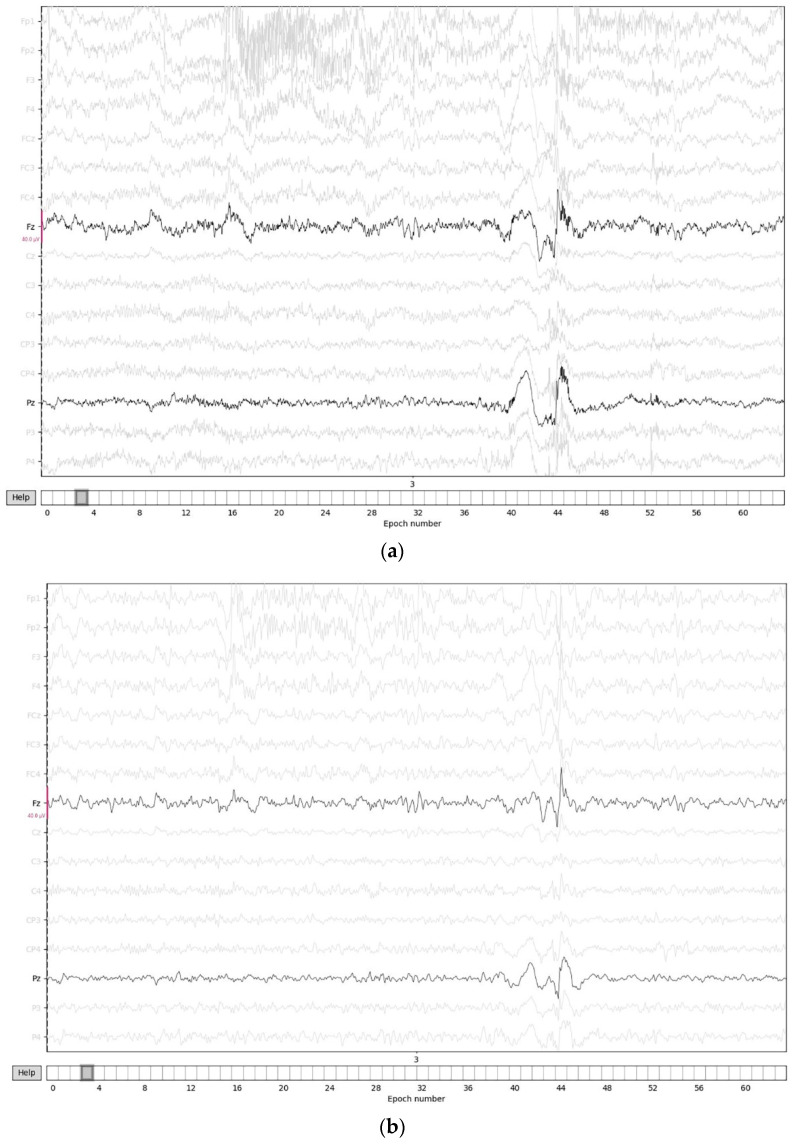

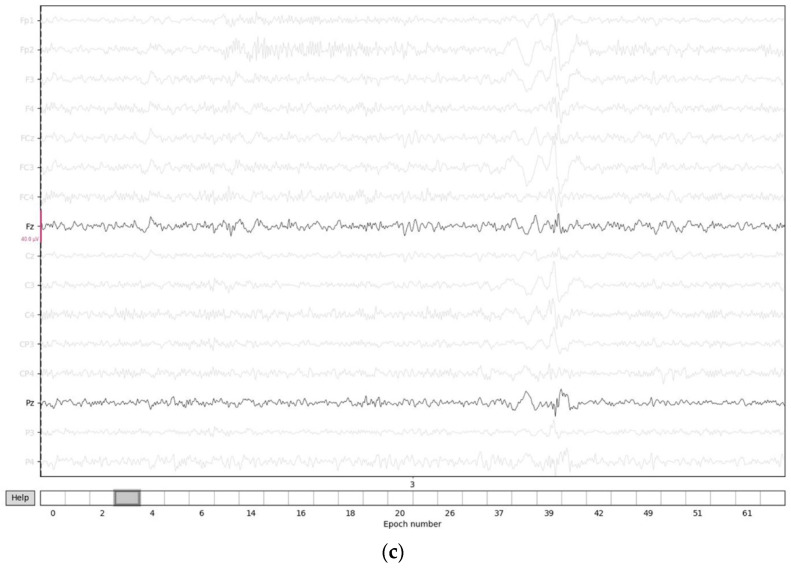
EEG signals: (**a**) raw EEG signals; (**b**) filtered EEG signals; (**c**) artifact-free EEG signals.

EEG data were preprocessed using MNE-Python, an open-source software package. Continuous EEG data were band-pass filtered between 3 and 30 Hz (4th order Butterworth FIR) with additional notch filters to reduce line noise (50 Hz). Independent components analysis (ICA) was used to eliminate artifacts and epochs with an absolute difference larger than 150 μV were also excluded as artifacts. The topographic map of ICA components is shown in Figure 1. The raw, filtered, and artifact-free EEG signals are shown in Figure 2a–c. Data were cut into 3.5 s epochs for drop-jump landing. In the EEG signals, Fast Fourier Transforms (FFT) were calculated. The average power spectra were computed for each trial in each subject and divided into different frequencies: theta (4–7 Hz), alpha-1 (7–10 Hz), and alpha-2 (10–12 Hz). Finally, theta and alpha (alpha-1 and alpha-2) activity in the frontal (Fz) and parietal (Pz) lobes were analyzed, respectively. 

### 2.5. Statistical Analysis

The data were analyzed using SPSS software version 24.0 (IBM Corp, Armonk, NY, USA). Shapiro–Wilk (S-W) test and Levene test were used to determine whether variables fitted normal distribution and variance homogeneity, respectively. The EEG outcome measures were transformed to normal distribution by using natural logarithmic based transformation, if needed. Continuous variables are reported as mean ± standard deviation. Demographics data between two groups were compared using independent-samples t test. To examine cortical activity a mixed-design ANOVA with 2 groups (CAI vs. CON) as between-subject factor, 2 limbs (injured vs. healthy) as within-subject factor was conducted. Partial eta squares (η^2^p) were reported to show the effect sizes. Effect sizes of <0.01 were interpreted as small, 0.01 to 0.06 as medium, and ≥0.14 as large effect [41]. The statistical significance level was set at *p* < 0.05.

## 3. Results

Brain activity demonstrated significantly higher frontal (Fz) theta power in the CAI group vs. the healthy control group (F_(1,22)_ = 7.726, *p* = 0.011, η^2^p = 0.260) (Table 2), indicating an increased cortical activation during drop-jump landing among CAI patients. There was no difference in alpha-1 and alpha-2 power in the parietal (Pz) cortex between the CAI and CON group (alpha-1: F_(1,22)_ = 0.297, *p* = 0.591, η^2^p = 0.013; alpha-2: F_(1,22)_ = 0.118, *p* = 0.734, η^2^p = 0.005) (Table 2). In addition, we did not find any significant differences in alpha-1, alpha-2, and theta power between the injured and healthy side in CAI patients (alpha-1: F_(1,22)_ = 0.149, *p* = 0.703, η^2^p = 0.007; alpha-2: F_(1,22)_ = 0.166, *p* = 0.688, η^2^p = 0.007; theta: F_(1,22)_ = 2.256, *p* = 0.147, η^2^p = 0.093) (Table 3).

## 4. Discussion

To investigate the activation differences on the cerebral cortex in people with chronic ankle instability, we measured and compared the EEG power at different frequencies during injury-related drop-jump landing movement in soccer athletes with and without CAI. Partially consistent with our research hypothesis, the theta band power was increased in soccer athletes with CAI during drop-jump landing, but there were no differences in alpha-1 and alpha-2 activity between groups or between limbs. 

The main findings in our study were that theta band power in the frontal area (Fz) showed significant difference between groups (CAI vs. CON), but we did not find any limb (injured vs. healthy) differences in the CAI group during drop-jump landing. Theta rhythm is a 4–8 Hz oscillation in the local field potential and has been linked to the encoding of new information into episodic memory and spatial navigation, which was first characterized in rodents in the 1930s [42]. Since theta power increases in a large variety of different tasks, it is reasonable to assume that theta power reflects unspecific factors such as task difficulty, attentional demands, and cognitive load [43]. The frontal theta frequency is thought to be generated in the anterior cingulate cortex which is an important part of the human attentional system involved in target selection, error detection, and performance monitoring [44]. In addition, increased frontal theta power indicates that individuals are engaged in a more complex and vigilant situation than that in a resting state [44]. Therefore, frontal theta power is frequently used as an indicator of concentration and task complexity. Consistent with our assumptions, soccer athletes with CAI demonstrated a significantly higher theta power on the Fz electrode, indicating a higher focused attention compared with the CON group. Speculations could lead to the idea that drop-jump landing from the same height makes the task more complex for CAI individuals, and therefore, a higher level of concentration is needed to select the relevant information to maintain stability during the drop-jump landing task. This result was supported by Baumeister, who found increased frontal theta power in ACL-reconstructed patients compared with the healthy control group while they were asked to reproduce knee angle [45]. In addition, Miao found that compared with healthy individuals, the theta band power significantly increased in ACL rupture participants during walking, jogging, and landing [46]. According to the neuroplasticity hypothesis, neural changes have been interpreted as adaptation strategies of the CNS to compensate for the trauma-induced sensory deafferentation aiming to maintain motor tasks performance [19]. However, Giesche [47] did not find any differences between the ACL-reconstructed and healthy control group in frontal theta power. This result may be due to the fact that he assessed the cortical activity before movement initiation, in which the subjects stood in a stable position without needing excessive sensory input from the knee joint. Unlike Giesche’s study, the trials in our experiment covered movement preparation, initiation, and execution; especially in patients with ankle instability, maintaining a stable landing can be challenging, which may make a difference between groups in frontal theta. Although the results mentioned above were derived from the knee joint, we can still obtain evidence about cortical activation alterations following ligament injury. To achieve joint stability, the peripheral mechanoreceptors such as ligament, cutaneous, and tendon would elicit action potentials along afferent axons to the spinal cord in the process of joint loading [33]. The sensory signal would then continue ascending in the central nervous system by passing through the thalamus and terminating in the primary somatosensory cortex [48]. Unfortunately, joint injury could result in a lack of neural communication between the ligament and the somatosensory cortex [32,49], which affects somatosensory perception in CAI patients [29]. As a result, individuals with CAI reflect the need for higher neurocognitive attention and processing during muscle contraction to compensate for the loss of sensory input from the injured ankle joint. Therefore, we believed that soccer athletes with CAI showed higher frontal theta power for movement planning and movement execution as compensation for reduced afferent information during the drop-jump landing task of the injured joint. 

We did not find any group (CAI vs. CON) or limb (injured vs. healthy) differences in parietal (Pz) alpha (alpha-1 and alpha-2) power during drop-jump landing among soccer athletes. Alpha is one of the fundamental functional operators of the brain for signal processing and communication in sensory or cognitive processes in the brain [50]. In addition, alpha activity is described as a form of cortical idling, with its amplitude inversely related to the amount of cortical resources allocated to task performance [51,52], showing relatively extensive event-related desynchronization (ERD) in perception, judgment, and memory tasks [53,54,55]. Previous studies have further divided alpha band into slow (alpha-1, 7–10 Hz) and fast (alpha-2, 10–12 Hz) components, and have found them to have slightly different task correlates [56]. Alpha-1 is obtained widespread over broad regions of the cortex and in response to almost any type of task and attentional process, with reduced power values indicating a global activation and therefore a higher unspecific arousal level [57]. Alpha-2 reflects restricted and task-specific demands and develops during the processing of sensory-semantic information in parieto-occipital regions [58]. We investigated the alpha-1 and alpha-2 power in the parietal (Pz) area among soccer athletes with and without CAI. Considering the alpha rhythm tends to decrease in amplitude as tasks become more difficult and more attention-demanding [59,60], we hypothesized that soccer athletes with CAI will show lower alpha power during drop-jump landing. However, no difference on parietal alpha-1 and alpha-2 power was found in the current study. Contrary to our findings, Baumeister [45] found decreased alpha-2 power in the parietal cortex in ACL reconstruction patients compared with healthy control individuals in a joint position paradigm, indicating that the patients potentially require more neuronal resources in the parietal cortex while performing the more complex task. Similar to Baumeister’s study, other researchers [44,61,62] have found increased alpha-2 power in experts or the intelligent compared with novices or the average. A higher level of alpha amplitude has been seen as an index of top-down processing, representing a mechanism for increasing the signal to noise ratio within the cortex by actively inhibiting non-essential or conflicting processes [63,64]. Effective cognition is not a function of how hard the brain works but rather how efficiently it works, which is known as the neural efficiency hypothesis. Training-related changes in alpha power might be interpreted as a strategy to use neuronal resources more efficiently. It has been reported that EEG activity in the alpha band increased with training in CAI patients [35]. In addition, Slobounov [40] revealed that depression of alpha power was associated with loss of balance and considered alpha activity as a predictor of future instability in posture. 

There could be several reasons why our results did not match the expectations. At first, there may be a ceiling effect in our study subjects. The ability to focus on sensory information related to performance is one of the key elements for elite athletes [65]. In addition, the CNS of soccer athletes has highly adapted due to long-term training and competition. Therefore, they had developed task solving strategies to achieve successful performance, such as focused attention and an economy in sensory information processing. This, combined with the small sample size of our preliminary study, makes it difficult to detect alpha (alpha-1 and alpha-2) power differences during drop-jump landing. In addition, the small number of movement repetitions is also a limitation of our research. We chose three times of successful completion of the task as the statistical calculation times of the EEG, which is supported by Slobounov and Miao [40,46]. However, considering that dynamic tasks tend to produce excessive noise and movement artifacts, a minimum of 40 trials per participant was recommended to obtain a steady EEG signal [34,66]. We will improve the testing paradigm in future studies. Finally, the amplitude of band activity is not only affected by age, mental state, and the cognitive task being performed, but also by the cerebral location from which the EEG signal is being recorded [50]. Therefore, signal acquisition at different electrodes may lead to inconsistent results. Burcal [34] measured the electrical activity of the alpha band on the Cz electrode and found that the amplitude of alpha activity did not change after 4 weeks of balance training in a CAI population. Uzlasir [35] found that after receiving balance training using stroboscopic glasses, the alpha power on the Cz electrode increased, while the alpha power on te occipital region remained unchanged in CAI patients. Moreover, Miao et al. [46] calculated alpha power on nine electrodes from the frontal area to parietal lobe, and also obtained inconsistent results. Basar once stressed that different locations in the brain may show completely different behaviors, and that might help to account for the discrepancies in the results of several authors by facilitating hypotheses based on alpha activity [50]. Based on the fact that correlations and coherences between all locations are functionally relevant, the view that assumptions about alpha activity should include all locations has been proposed. 

It is also worth noting that we did not find any differences in alpha (alpha-1 and alpha-2) and theta powers between the involved and uninvolved side in CAI patients, which means that we could observe EEG changes during the drop-jump landing task in CAI patients even on their uninvolved side. The result is in line with earlier findings reported by other authors. In an investigation to evaluate corticomotor excitability in individuals with CAI, Pietrosimone [21] and McLeod [22] separately demonstrated that the CAI group had higher bilateral fibularis longus resting motor threshold and lower bilateral fibularis longus motor-evoked potentials amplitudes compared with healthy controls, indicating decreased descending corticomotor excitability of bilateral fibularis longus muscles which can stabilize the ankle joint. A study on EEG after ligament injury by Baumeister [45] observed that ACL reconstruction also influences the uninvolved side. These bilateral motor cortical excitability changes after unilateral joint injury suggest that functional reorganization of the CNS overrides mechanical deficits. Thus, the idea of using the uninjured side as a reference should be seriously reconsidered. 

There are also some limitations to our study. Firstly, the retrospective study design was unable to determine if the changes in power spectrum occur after unilateral CAI or if the altered cortical activation is a predisposing factor that may lead to CAI. That is, we cannot establish a causal relationship between CAI and cortical changes. Therefore, prospective investigations are needed to verify our speculation. Secondly, although our study investigated the cortical alterations in more sport-relevant and injury-relevant patterns compared with studies assessing in sitting or lying position, we still chose the anticipated task mainly requiring feedback control. Considering athletes are required to quickly adapt to changing environments and cannot just rely on anticipated movements, future studies should include unanticipated jump-landing tasks in order to elucidate its value in predicting injury and monitoring recovery. Thirdly, due to the COVID-19 prevention and control requirements of Beijing Sport University and considering that this experiment is a preliminary study, we failed to recruit enough healthy control subjects, which may have masked cortical differences that could be identified with larger sample sizes. Therefore, we suggest that at least 16 participants be included in each group in future study (power = 0.8, effect size = 0.4). Finally, only three trials per participant were required for analysis, and it was difficult to obtain stable EEG signals. Thus, researchers should reconsider the number of tests based on different research objectives. 

## 5. Conclusions

Theta power at the frontal cortex was higher in soccer athletes with CAI than in healthy controls during drop-jump landing. However, no differences existed in parietal alpha-1 and alpha-2 power between groups. We did not find differences in alpha and theta power between the injured and healthy side in soccer athletes with CAI.

## Figures and Tables

**Table 1:** Participant Demographics.

Measure	CAI	CON	t	*p*
Gender, *n* (M/F)	13/3	6/2		
Age, y	20.81 ± 1.56	20.38 ± 1.19	0.696	0.493
Height, cm	175.41 ± 8.54	176.00 ± 6.16	−0.173	0.865
Mass, kg	70.58 ± 9.34	66.15 ± 8.87	1.112	0.278
CAIT, score	18.31 ± 2.52	30.00 ± 0.00	−18.534	0.000 *

CAI: chronic ankle instability group; CON: healthy control group; M: male; F: female; CAIT: Cumberland Ankle Instability Tool. * Indicates *p* < 0.05.

**Table 2:** EEG results in CAI and CON groups.

	Drop-Jump Landing				
Measure	CAI	CON	F	*p*	η^2^p
Alpha-1 (Pz)	2.84 ± 1.05	3.10 ± 1.32	0.297	0.591	0.013
Alpha-2 (Pz)	2.54 ± 1.09	2.71 ± 1.16	0.118	0.734	0.005
Theta (Fz)	4.27 ± 0.73	3.47 ± 0.50	7.726	0.011 *	0.260

CAI: chronic ankle instability group; CON: healthy control group; * indicates *p* < 0.05.

**Table 3:** EEG results between two limbs in CAI group.

	CAI				
Measure	Injured Side	Healthy Side	F	*p*	η^2^p
Alpha-1 (Pz)	2.84 ± 1.05	2.92 ± 0.92	0.149	0.703	0.007
Alpha-2 (Pz)	2.54 ± 1.09	2.61 ± 1.01	0.166	0.688	0.007
Theta (Fz)	4.27 ± 0.73	4.06 ± 0.81	2.256	0.147	0.093

CAI: chronic ankle instability group.

## Data Availability

The data presented in this study are available on request from the corresponding author.
